# Biochemical Hypogonadism in Aging Testicular Cancer Survivors: A Clinical Challenge

**DOI:** 10.1016/j.euros.2024.12.010

**Published:** 2025-01-17

**Authors:** Sophie D. Fosså, Lars J. Bjerner, Torgrim Tandstad, Marianne Brydøy, Alv A. Dahl, Ragnhild V. Nome, Helene Negaard, Tor Å. Myklebust, Hege S. Haugnes

**Affiliations:** a Department of Oncology, Oslo University Hospital, Oslo, Norway; b Institute of Clinical Medicine, University of Oslo, Oslo, Norway; c Fürst Medical Laboratory, Oslo, Norway; d Cancer Clinic, St. Olavs University Hospital, Trondheim, Norway; e Department of Clinical and Molecular Medicine, Norwegian University of Science and Technology, Trondheim, Norway; f Department of Oncology and Medical Physics, Haukeland University Hospital, Bergen, Norway; g Department of Medical Biochemistry, Oslo University Hospital, Norway; h Department of Registration, Cancer Registry of Norway, Oslo, Norway; i Research and Innovation, Møre and Romsdal Hospital Trust, Ålesund, Norway; j Department of Oncology, University Hospital of North Norway, Tromsø, Norway; k Institute of Clinical Medicine, Arctic University, Tromsø, Norway

**Keywords:** Testicular cancer, Survivors, Biochemical hypogonadism, Change, Adverse health outcomes

## Abstract

**Background and objective:**

Few longitudinal studies have described the prevalence and development of biochemical hypogonadism in aging testicular cancer survivors (TCSs) in comparison to men from the general population (control subjects).

**Methods:**

Serum total and free testosterone (T_total_, T_free_) were measured in 593 TCSs median11 and 27 years after TC diagnosis (Survey-First; Survey-Last). Post-treatment adverse health outcomes (AHOs) were recorded. The results were compared to those in 578 control subjects. Treatment was stratified as surgery alone, radiotherapy alone, or platinum-based chemotherapy. Biochemical hypogonadism was defined as T_total_ <8 nmol/l, or as T_total_ <12 nmol/l and T_free_ <225 pmol/l. We used multivariable logistic regression analysis to explore associations with age and treatment intensity. Statistical significance was set at *p* <0.05.

**Key findings and limitations:**

Between the first and last survey the prevalence of biochemical hypogonadism increased from 12% to 41% in the TSC group and from 5% to 11% in the control group. Three decades after diagnosis, the probability of biochemical hypogonadism was significantly correlated with increasing age and greater treatment intensity. The combined age- and treatment- related probability of hypogonadism was more than threefold higher in the TCS group than in the control group. At the last survey, fewer eugonadal than hypogonadal TCS men reported at least one AHO attributable to androgen deficiency (54% vs 72%; *p* <0.001). Limitations include the availability of only one blood sample per survey wave.

**Conclusions and clinical implications:**

For aging TCSs, the probability of biochemical hypogonadism depends on age and prior treatment intensity and is threefold higher than for control subjects at 30 yr after diagnosis. As late hypogonadism is associated with AHO incidence, the development of hypogonadism should be monitored via regular blood tests during TCS follow-up.

**Patient summary:**

Depending on the treatment they received, older survivors of testicular cancer (TC) are at persistent risk of lower testosterone levels. Our study revealed low testosterone in 40% of TC survivors older than 60 years compared to 10% of similarly aged men from the general population. Low testosterone is associated with chronic conditions such as diabetes, fatigue, and/or erectile dysfunction. Testosterone should be regularly monitored during follow-up for TC survivors.

## Take Home Message

Biochemical hypogonadism (BCH) is three to four times more prevalent among testicular cancer survivors aged >60 yr than among control subjects. BCH is associated with the development of chronic morbidity typical in elderly males. Serum testosterone should be regularly monitored during follow-up for testicular cancer survivors.

## 1 Introduction

It has been reported that late biochemical hypogonadism (BCH) involving low serum levels of total testosterone (T_total_) and free testosterone (T_free_) occurs in 2–12% of men aged 40–70 years in the general population [1], [2], [3], [4], [5]. The presence of symptoms attributable to androgen deficiency separates clinical hypogonadism from BCH [6]. BCH represents a clinical challenge, as it is positively associated with the development of chronic adverse health outcomes (AHOs) such as cardiovascular morbidity, diabetes, fatigue, and sexual dysfunction [5], [7].

In comparison to similarly aged men in the general population, aging testicular cancer survivors (TCSs) have a higher risk of AHOs [8], [9], [10], [11], [12] that depends on treatment intensity and may be associated with androgen deficiency. In cross-sectional studies, androgen deficiency has been observed in up to 40% of TCSs, most of them aged <50 yr [13], [14], [15].

The focus of the current study is on longitudinal development of late hypogonadism in aging TCSs, applying the European definitions of hypogonadism [16], [17]. We asked the following research questions: (1) How does the prevalence of late BCH and the probability of its development differ between aging TCSs and similarly aged men from the general population? (2) Are there any factors associated with the probability of hypogonadism in aging TCSs? We also explored the associations between BCH and selected post-treatment AHOs attributable to androgen deficiency.

## 2 Patients and methods

### 2.1 Patients

From 1998 to 2017, a three-wave national survey explored post-treatment AHOs in a TCS cohort of men who underwent unilateral orchiectomy in Norway between 1980 and 1994 [10], [18]. In each survey wave, TCS respondents completed a questionnaire describing their AHOs und underwent a clinical examination and routine blood tests in blood sampled before noon. Residual serum was deep-frozen at −70 °C.

Here we report analyses for 593 TCSs for whom deep-frozen sera from the first and third survey waves (first survey and last survey) were available (Supplementary Fig. 1). TCSs on androgen replacement therapy (ART) at the first survey and those with suspected ART (T_total_ >35 nmol/l, follicle-stimulating hormone ≤1 U/l, and/or luteinizing hormone ≤1 U/l) were excluded from all analyses (*n* = 20), as well as men with a prostate cancer diagnosis (*n* = 20) as their treatment was unknown.

For each survey wave, the number of TCSs reporting three selected AHOs probably related to androgen deficiency, namely metabolic syndrome, sexual dysfunction, and fatigue, was assessed (Supplementary material). The number of TCSs with at least one AHO was calculated.

### 2.2 TC treatment

The treatment guidelines recommended in Norway from 1980 to 1994 are listed in Supplementary Table 1. The TCS group was categorized into four post-orchiectomy treatment subgroups according to combined initial and relapse treatment modalities.

­ Local treatment alone
  o Surgery alone: post-orchiectomy surveillance or diagnostic retroperitoneal lymph node dissection
  o -Radiotherapy alone
­ Systemic treatment (with or without local treatment)
  o Standard platinum-based chemotherapy (PBCT) with a cumulative platinum dose of ≤850 mg (≤4 PBCT cycles)
  o Nonstandard PBCT: a cumulative platinum dose of >850 mg or any number of PBCT cycles if combined with radiotherapy.

The term *cytotoxic treatment* covered both radiotherapy alone and all PBCT regimens.

### 2.3 Biochemical analyses

Thawed residual serum samples were analyzed for T_total_ (nmol/l), albumin, and sex hormone–binding globulin (SHBG) on a Roche Cobas 8000 system (Roche Diagnostics, Mannheim, Germany). Free testosterone (T_free_, pmol/L) was calculated using available data for T_total_, albumin, and SHBG levels [19]. Assays were subject to custom internal and external quality control measures (data not shown).

In accordance with the European Male Aging Study, BCH was defined as serum T_total_ <8 nmol/l, or as T_total_ 8–12 nmol/l and T_free_ <225 pmol/l [5], [16], [17]. All other test results corresponded to eugonadism.

### 2.4 Control group

After age adjustment according to the age of the TCS participants at the first and last surveys, evaluable control subjects were identified among men in the general population who had participated in the cross-sectional NORIP project [20]. No information about symptoms or diseases was available for the control subjects.

### 2.5 Statistical analysis

Results are presented as the absolute and relative frequencies for categorical variables, and as the median and interquartile range, or mean and standard deviation for continuous variables. Student’s t tests, one-way analysis of variance, and χ^2^ tests evaluated differences between groups. When comparing results between the TCSs and control groups, estimates for the control groups were age-standardized to the age distribution of the respective patient group via relative weighting of estimates for each of the age groups. Scatter plots showing the association between T_total_ and T_free_ levels and age were generated, including a fitted line using locally weighted smoothing. Multivariable logistic regression analyses (MVAs) were used to estimate potential associations between selected covariates and the risk of BCH at the first and last surveys. Covariates included age at survey and treatment modalities, and a dichotomous covariate for the last survey indicating whether BCH had been present at the first survey.

Age at survey wave was modeled using restricted cubic splines with knots placed at the 5th, 35th, 65th, and 95th centiles of the age distributions. Predicted probabilities at set values of age and treatment were calculated from the fitted models and were presented graphically. As testing did not support significant nonlinearity, odds ratios (ORs) were presented by 10-yr increments in age. A *p* value <0.05 was considered statistically significant, and tests were two-sided. Analyses were conducted using SPSS v29 and Stata v18.0.

### 2.6 Ethics

The study was approved by the Committee for Medical Research Ethics of the Southern Health Region of Norway (2015/1264). All participants provided written informed consent.

## 3 Results

### 3.1 Study cohort

Median age was similar for the group of 593 evaluable TCSs and the 597 patients excluded (data not shown). Median follow-up time from diagnosis was 11 yr for the first survey and 27 yr for the last survey (Table 1), with corresponding increases in median age of the study participants over the observation period of 16 yr.

**Table 1:** Demographics and treatment details for the 593 testicular cancer survivors

Parameter	Result
Year of diagnosis, *n* (%)	
1980–1984	147 (25)
1985–1989	174 (29)
1990–1994	272 (46)
Median follow-up since diagnosis, yr (IQR)	
To first survey	11 (5–16)
To last survey	27 (24–31)
Median age at diagnosis, yr (IQR)	31 (26;36)
Median age at survey wave, yr (IQR)	
First survey	43 (38–50)
Last survey	59 (54–66)
Seminoma histology, *n* (%)	279 (47)
Treatment, *n* (%)	
Surgery only	120 (20)
Surveillance	52
RPLND	68
Radiotherapy only	232 (40)
Dogleg field	222
Para-aortic strip	10
Standard PBCT	177 (30)
≤3 cycles	65
4 cycles	112
Nonstandard PBCT	64 (11)
>4 PBCT cycles	34
PBCT + radiotherapy	30

IQR = interquartile range; PBCT = platinum-based chemotherapy.

### 3.2 Testosterone levels

At the first survey there were larger differences in T_total_ and T_free_ across age groups than across treatment groups, suggesting a somewhat stronger association between testosterone levels and age than between testosterone levels and treatment (Table 2). At the last survey the corresponding differences were of the same order of magnitude. The largest treatment-related reduction in both T_total_ and T_free_ was observed for patients with prior nonstandard PBCT. Supplementary Fig. 2 shows that age was more strongly associated with T_free_ than with T_total_.

**Table 2:** Median total and free testosterone levels by survey wave with stratification by age at first survey and treatment among 593 testicular cancer survivors a

	First survey	Last survey	*p* value
**Total testosterone (nmol/l)**			
Overall	14.9 (12.1–18.3)	12.5 (9.7–16.2)	<0.001
By age category at first survey			
<40 yr	15.6 (12.6–19.0)	13.1 (10.0–16.4) b	
40–60 yr	14.7 (11.8–18.2)	12.5 (9.7–16.1)	
>60 yr	13.2 (9.5–16.2)	9.8 (6.7–15.9)	
By treatment category			
Surgery only	15.8 (12.5–19.6)	14.1 (11.5–17.6)	
Radiotherapy only	14.6 (12.0–17.8)	12.3 (9.8–15.3)	
Standard platinum-based chemotherapy	14.8 (11.7–18.4)	12.9 (9.4–17.0)	
Nonstandard platinum-based chemotherapy	14.7 (11.0–17.1)	10.6 (7.5–13.9)	
**Free testosterone (pmol/l)**			
Overall	310 (253–379)	209 (163–254)	<0.001
By age category at first survey			
<40 yr	338 (280–403)	232 (182–273)	
40–60 yr	293 (242–363)	202 (155–202)	
>60 yr	234 (192–279)	148 (86–190)	
By treatment category			
Surgery only	324 (266–363)	228 (186–264)	
Radiotherapy only	308 (247–378)	207 (160–249)	
Standard platinum-based chemotherapy	318 (252–382)	211 (173–262)	
Nonstandard platinum-based chemotherapy	291 (246–384)	169 (127–236)	

a Results are reported as median (interquartile range).

b Only three participants were evaluable.

### 3.3 Biochemical hypogonadism

Over the observation period, BCH prevalence tripled from 12% at the first survey to 41% at the last survey in the TCS cohort, and doubled from 5% to 11% in the control group (Table 3). At the first survey, BCH prevalence was higher in the radiotherapy alone and both PBCT groups than in the surgery alone and control groups. At the last survey, these treatment-related differences between the TCS and control groups had increased. Subgroup analyses at the last survey revealed BCH prevalence of 42% after three PBCT cycles and 47% after four cycles, but the difference did not reach statistical significance. Notably, the BCH prevalence at the first survey was similar between the surgery alone (7%) and control (5%) groups. Three decades after diagnosis, the BCH rate was 25% for the surgery alone group and just but only 11% in the control group (Table 3).

**Table 3:** Prevalence of biochemical hypogonadism in the control group (*n* = 578) and the TCS group (*n* = 593) and stratified by overall treatment

Treatment	Biochemical hypogonadism, *n* (%)			
	First survey		Last survey	
	TCS group	Control group	TCS group	Control group
Overall	73 (12)	30 (5)	242 (41)	61 (11)
Surgery only (*n* = 120)	8 (7)		30 (25)	
Radiotherapy only (*n* = 232)	31 (13)		98 (42)	
Standard PBCT (*n* = 177)	24 (14)		76 (43)	
Nonstandard PBCT (*n* = 64)	10 (16)		38 (59)	

PBCT = platinum-based chemotherapy; TCS = testicular cancer survivor.

Table 4 lists T_total_ and T_free_ levels and AHO prevalence at each survey wave stratified by hypogonadal versus eugonadal status. Among the TCS groups the prevalence of each of the three selected AHOs doubled between the two surveys. At the last survey, the occurrence of at least one AHO was reported by 72% ofthe hypogonadal TCSs versus 54% of the eugonadal TCSs (*p* <0.001).

**Table 4:** Total and free testosterone levels and selected AHOs stratified by eugonadism versus hypogonadism status a

Parameter	Testicular cancer survivors (*n* = 593)		Control group (*n* = 571)	
	First survey	Last survey	First survey b	Last survey b
**Hypogonadism**				
Participants, *n* (%)	73 (12%)	242 (41%)	30 (5)	61 (11%)
Age at survey wave (yr)	48 (43–53)	61 (55–68)	54 (44–57)	58 (54–64)
Total testosterone (nmol/l)	8.8 (7.3–10.4)	9.0 (6.9–10.6)	7.4 (1.6–9.7)	6.9 (6.7–10.5)
Free testosterone (pmol/l)	193 (166–211)	158 (119–185)	144 (20–176)	171 (96–196)
AHOs, *n* (%)				
Metabolic syndrome	18 (25%)	124 (51%)		
Sexual dysfunction	9 (12%)	87 (36%)		
Fatigue	11 (15%)	64 (26%)		
≥1 AHO	31 (43%)	173 (72%)		
**Eugonadism**				
Participants, *n* (%)	520 (88)	351 (59)	541 (95)	513 (89)
Age at survey (yr)	42 (37–49)	58 (53–64)	43 (35–50)	57 (52–63)
Total testosterone (nmol/l)	15.7 (13.1–18.7)	15.2 (13.3–18.6)	18.7 (15–22)	18.2 (14.5–21.6)
Free testosterone (pmol/l)	328 (275–385)	246 (219–282)	378 (304–441)	321 (270–388)
AHOs, *n* (%)				
Metabolic syndrome	80 (15%)	110 (32%)		
Sexual dysfunction	60 (12%)	82 (23%)		
Fatigue	77 (15%)	78 (23%)		
≥1 AHO	174 (34%)	189 (54%)		

AHO = adverse health outcome

a Results for continuous variables are reported as median (interquartile range)

b After age adjustment on the basis of age in the testicular cancer survivor group at the first or last survey.

At the first survey, increasing age and treatment intensity were significantly associated with higher probability of BCH (Table 5, model A). In comparison to surgery alone, the ORs were significantly higher for standard and nonstandard PBCT. At the last survey (Table 5, model B1) both age and treatment intensity were significantly associated with the risk of late BCH (*p* <0.001). Three decades after treatment, the risk of BCH doubled after radiotherapy alone and standard PBCT, and was almost fourfold higher after nonstandard PBCT in comparison to surgery alone. The highest OR at the last survey (4.85, 95% confidence interval 2.69–8.73) was observed when BCH at the first survey was included as a covariate (Table 5, model B2).

**Table 5:** Logistic regression results for biochemical hypogonadism as the outcome

Parameter	First survey		Last survey			
	Model A a		Model B1 a		Model B2 b	
	OR (95% CI)	*p* value	OR (95% CI)	*p* value	OR (95% CI)	*p* value
Age at survey per 10-yr increment	2.02 (1.71–2.39) c	<0.001	1.55 (1.27–1.88) d	<0.001	1.36 (1.11–1.68) d	0.004
Treatment		0.012		<0.001		<0.001
Surgery only	Reference		Reference		Reference	
Radiotherapy only	1.46 (0.82–2.58)		1.72 (1.06–2.78)		1.84 (1.11–3.07)	
Standard PBCT	2.13 (1.16–3.91)		2.16 (1.31–3.56)		2.16 (1.28–3.68)	
Nonstandard PBCT	3.32 (1.71–6.43)		3.71 (1.96–7.02)		4.33 (2.21–8.50)	
BCH at first survey (yes vs no)					4.85 (2.69–8.73)	<0.001

BCH = biochemical hypogonadism; CI = confidence interval; OR = odds ratio; PBCT = platinum-based chemotherapy.

a Age at survey wave and treatment as independent variables.

b Age at last survey, treatment, and BCH at first survey as independent variables.

c Test for nonlinear trend: *p* = 0.39.

d Test for nonlinear trend: *p* = 0.94.

### 3.4 Combined effect of treatment and age

**Fig. 1:**
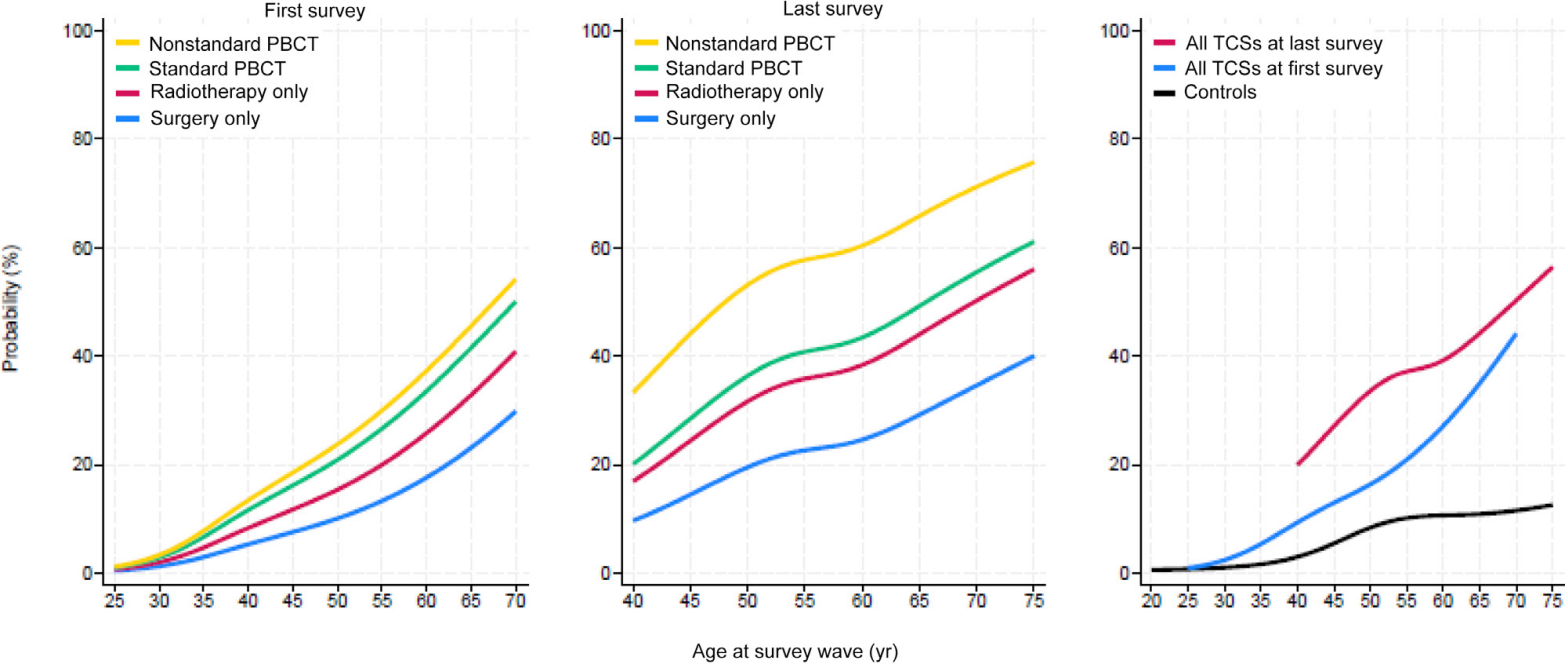
Probability of biochemical hypogonadism for testicular cancer survivors (TCSs) at the first and last surveys in comparison to the control group by age at survey wave, with stratification for treatment intensity. PBCT = platinum-based chemotherapy.

Fig. 1 shows the combined age- and treatment-related probability of BCH in the TCS cohort, and the age-dependent risk in the control group. In comparison to the control group, the probability of BCH increased with age at both survey waves. Overall, the probability of BCH was more than threefold higher for the TCS cohort than for the control group. At the last survey, the risk of BCH was highest in the nonstandard PBCT group, and was similar in the radiotherapy alone and standard PBCT groups.

## 4 Discussion

Our longitudinal study including a control group from the general population is the first to document the rising probability of late BCH in aging TCSs. At the first survey (median of 11 years after diagnosis, median age 43 yr), older age and prior treatment, especially PBCT, in comparison to surgery alone were significantly associated with BCH. At the last survey (median 27 yr after diagnosis), BCH prevalence was associated with age and increasing prior treatment intensity. Three decades after the TC diagnosis, the BCH rate was 41% in the TCS cohort versus 11% in the control group. At this time point, 72% of the hypogonadal and 54% of the eugonadal TCS subgroups reported at least one post-treatment AHO attributable to androgen deficiency (*p* <0.001).

### 4.1 Criteria

The prevalence of BCH in our control group is similar to results for elderly males in the US general population [1], which supports the methodology used for our analyses. Our study is based on the European criteria for BCH [5], [17] using just T_free_ and T_total_ and do not take symptoms compatible with androgen deficiency into account [6].

### 4.2 BCH development and prevalence

Published prevalence rates for BCH and/or androgen deficiency vary between 20% and 40%, and in some cases include TCSs using ART or those with elevated gonadotropin levels [13], [14]. Varying definitions of hypogonadism and differences regarding the length of post-treatment observation times and/or the age profile of TCS cohorts are some, but not all of, the reasons for the differences in prevalence rates. Notably, most studies on hypogonadism among TCSs were performed up to 10 years after diagnosis, whereas we found that the prevalence of hypogonadism increased with the post-treatment observation time beyond the first decade after diagnosis. Furthermore, in most of the studies, TCSs after surgery alone were used as the reference group, which is questionable. In comparison to our control group, the prevalence of hypogonadism was at the last survey significantly higher in the TCS surgery alone group , indicating late Leydig cell dysfunction in the remaining testicle.

The current study supports the view of Sprauten et al [15] on “premature hormonal aging” in TCSs and confirms the higher prevalence of androgen deficiency after cytotoxic treatment revealed by a meta-analysis [13]. However, the rather similar prevalence and risk data for the radiotherapy alone and standard PBCT groups at the last survey are somewhat surprising. Age differences at treatment may explain the limited difference between these treatment groups. Radiotherapy alone was the routine treatment modality for patients with nonmetastatic seminoma in our cohort, who were generally older at diagnosis than patients with metastatic nonseminoma, whose treatment mainly consisted of standard PBCT. High age is generally associated with greater vulnerability of Leydig cells and a lower capacity for postdamage recovery [21]. The threefold higher probability of BCH among TCSs requires further explanations beyond prior cytotoxic treatment. A negative impact of TC treatment on the hypothalamic-pituitary-gonadal axis and an increasing age-dependent impact of testicular dysgenesis syndrome in the remaining testis [22] should be considered.

### 4.3 Clinical implications

A BCH result for a single blood sample from a TCS does not mean that ART should be started immediately. Confirmation via repeat blood tests supplemented by determination of gonadotropin levels is necessary and can exclude any spontaneous reversal of low testosterone levels. According to the US [6] and European guidelines [14], [16], new serum testosterone tests should be performed and clinical hypogonadism should be excluded. If BCH persists despite lifestyle changes (weight reduction, increased physical activity, smoking cessation), ART should be considered in cases of co-existent comorbidity attributable to androgen deficiency [23], [24].

Our MVA results indicate that if BCH is detected a decade after TC diagnosis, health professionals, esponsible for long-term follow-up of TCSs, should be made aware of the higher probability of future long-lasting androgen deficiency. Even in asymptomatic TCSs, serum T_total_ should be determined during the first years of follow-up to establish the baseline situation. Alternatively, T_total_ should be measured in all TCSs when routine contact with the uro-oncologist is discontinued 5–10 yr after TC treatment [25]. In cases of confirmed BCH, the individual TCS and his general physician should be informed about the higher long-term probability of symptomatic clinical hypogonadism in order to plan an individualized follow-up schedule.

### 4.4 Limitations and strengths

Several limitations should be noted. First, BCH prevalence rates in our study are based on single blood samples analyzed with immunoassays, for which the diagnostic accuracy is a matter of debate [26]. Second, we did not distinguish between primary and secondary hypogonadism on the basis of gonadotropin levels, which will be addressed in a future project. Third, we included treatment regimens that are no longer consistent with current routine practice for contemporary TC patients, such as radiotherapy and nonstandard PBCT. However, radiotherapy is still frequently used for seminoma in low- and middle-income countries [27]. Furthermore, many elderly TCSs treated with these unconventional regimens are still alive in Europe and the USA and deserve optimal follow-up care, so awareness of the risk of late hypogonadism among physicians is important. Finally, no longitudinal blood samples were available for the control group and the BCH results are based on age-adjusted analyses. The large national TCS cohort that has been followed longitudinally for almost 30 years since diagnosis and the use of internationally accepted BCH criteria are the main strengths of the study.

## 5 Conclusions

In comparison to similarly aged men in the general population, elderly TCSs have an at least threefold higher probability of late BCH. Increasing age and the intensity of prior treatment continuously increase the probability of hypogonadism for up to three decades after TC diagnosis. Awareness of this issue allows appropriate planning of risk-adapted follow-up for long-term TCSs. To reduce the probability of symptomatic clinical hypogonadism, serum testosterone should be analyzed at the last consultation with the uro-oncologist. If BCH is detected at that time, the patient and his general practitioner should be informed about the need for repeated testosterone measurement during long-term follow-up to reduce the risk of AHOs attributable to androgen deficiency.

  ***Author contributions***: Sophie D. Fosså had full access to all the data in the study and takes responsibility for the integrity of the data and the accuracy of the data analysis.

  *Study concept and design*: Fosså, Haugnes, Myklebust, Bjerner.

*Acquisition of data*: Tandstad, Dahl. Brydøy.

*Analysis and interpretation of data*: Nome, Haugnes, Myklebust, Negaard.

*Drafting of the manuscript*: Fosså, Haugnes, Myklebust.

*Critical revision of the manuscript for important intellectual content*: Bjerner, Haugnes, Myklebust.

*Statistical analysis*: Myklebust, Fosså.

*Obtaining funding*: Fosså.

*Administrative, technical, or material support*: Fosså.

*Supervision*: Fosså.

*Other*: None.

  ***Financial disclosures:*** Sophie D. Fosså certifies that all conflicts of interest, including specific financial interests and relationships and affiliations relevant to the subject matter or materials discussed in the manuscript (eg, employment/affiliation, grants or funding, consultancies, honoraria, stock ownership or options, expert testimony, royalties, or patents filed, received, or pending), are the following: None.

  ***Funding/Support and role of the sponsor*:** This work was supported by the Radium Hospital Foundation under grant number 335007. The sponsor played a role in collection and analysis of the data.

  ***Acknowledgments*:** We are grateful for the valuable comments from endocrinologist Ansgar Heck and technical support from Vigdis Opperud and Siri Lothe Hess.
