# Supplementary material for: Biochemical Hypogonadism in Aging Testicular Cancer Survivors: A Clinical Challenge
Source: Eur Urol Open Sci. 2025 Jan 17;72:10–6. doi: 10.1016/j.euros.2024.12.010 (PMC11786855; doi:10.1016/j.euros.2024.12.010)
Supplement: Supplementary Data 1 [file mmc1.docx]

**Supplementary material**

**Definitions of Adverse Health Outcomes (AHOS) (“Caseness”)**

**Sexual dysfunction**

*Survey-Firs*t: Unfavorable response to question 8 and/or 9 of the Brief Sexual Function Inventory^1^.

Question 8: D*uring the past 30 days how would you rate your level of sexual drive?*  Unfavorable response: Medium or High problem

AND/OR

Question 9: *In the past 30 days, to what extent have you considered your ability to get and keep an erection a problem?* Unfavorable response; Big or Medium problem

*Survey-Last: Reported u*se of phosphodiesterase-5 inhibitors

and/or

Unfavorable response to item 12 of EPIC-26^2^: *Overall, how big a problem has your sexual function or lack of sexual function been for you during the last 4 weeks?* Unfavorable response: Moderate or Big problem

**Metabolic syndrome**

Report of ≥2 of the following conditions: Obesity (BMI > 30 kg/m^2^), Serum cholesterol ≥5.2 mmol/l or anti-lipid medication, anti-hypertension medication^3^, diabetes or use of antidiabetic medication

**Chronic Fatigue (CF):**

Assessmen**t** of physical and mental fatigue by finally dichotomized responses ( 0 versus 1) to the 11 questions of the Fatigue Questionnaire^4^ . A final scoring sum of ≥ 4 and duration of symptoms for at least 6 months identify a case of CF.

A missing response to a single item/question was counted as 0.

References

1. O'Leary MP, Fowler FJ, Lenderking WR, Barber B, Sagnier PP, Guess HA, Barry MJ. A brief male sexual function inventory for urology. Urology. 1995 Nov;46(5):697-706. doi: 10.1016/S0090-4295(99)80304-5. PMID: 7495124.
2. Szymanski KM, Wei JT, Dunn RL, Sanda MG. Development and validation of an abbreviated version of the expanded prostate cancer index composite instrument for measuring health-related quality of life among prostate cancer survivors. Urology. 2010 Nov;76(5):1245-50. doi: 10.1016/j.urology.2010.01.027. Epub 2010 Mar 28. PMID: 20350762; PMCID: PMC3152317.
3. Haugnes HS, Aass N, Fosså SD, Dahl O, Klepp O, Wist EA, Svartberg J, Wilsgaard T, Bremnes RM. Components of the metabolic syndrome in long-term survivors of testicular cancer. Ann Oncol. 2007 Feb;18(2):241-8. doi: 10.1093/annonc/mdl372. Epub 2006 Oct 23. PMID: 17060482.

**Supplementary Table 1: Primary treatment of TC in Norway (1980-1994)**

|  | **Non-metastatic disease^1^** | **Metastatic disease^1^** |
| --- | --- | --- |
| **A: Seminoma**  1980-1994 | Abdominal radiotherapy  40 → 30 Gy; Dogleg field / para-aortic strip^2^ | PBCT: 2-4 cycles followed by radiotherapy, surgery or observation only^3^ |
| **B: Non-seminoma**  1980 to 1989 | Non-seminoma: staging RPLND followed by adjuvant PBCT (2-3 cycles) if metastases were histologically verified ^4,5^ | PBCT as standard: CVB until 1987^6^ with BEP thereafter (4 cycles; 5 days/cycle). In selected patients at one institution experimental regimens within international research protocols.^7-11^  Post-PBCT surgery if residual masses. |
| 1990 to 1994 | Non-seminomas: RPLND was gradually replaced by surveillance or 1-2 cycles of adjuvant PBCT.^12,13^ | BEP-regimen (5 days cycles) as standard first-line therapy (low risk) or 4 (medium/high risk) cycles or participation in international trial ^7-11^ Post-PBCT surgery in case of residual masses. |
| ^1^Based on the Royal Marsden Hospital staging system  TC: Testicular Cancer; RPLND: retroperitoneal lymph node dissection; PBCT: cisPlatinum-based chemotherapy; CVB: Cisplatin, Vinblastine, Bleomycin; BEP: cisPlatinum, Etoposide, Bleomycin. | | |

**References**

1. Peckham MJ, McElwain TJ, Barrett A, et al: Combined management of malignant teratoma of the testis. Lancet 267-270, 1979.
2. Fosså SD, Horwich A, Russell JM, Roberts JT, Cullen MH, Hodson NJ, Jones WG, Yosef H, Duchesne GM, Owen JR, Grosch EJ, Chetiyawardana AD, Reed NS, Widmer B, Stenning SP. Optimal planning target volume for stage I testicular seminoma: A Medical Research Council randomized trial. Medical Research Council Testicular Tumor Working Group. J Clin Oncol. 1999 Apr;17(4):1146. doi: 10.1200/JCO.1999.17.4.1146. PMID: 10561173.
3. Fosså SD, Kullmann G, Lien HH, Stenwig AE, Ous S. Chemotherapy of advanced seminoma: clinical significance of radiological findings before and after treatment. Br J Urol. 1989 Nov;64(5):530-4. doi: 10.1111/j.1464-410x.1989.tb05293.x. PMID: 2611626.
4. Fosså SD, Klepp O, Ous S, et al: Unilateral retroperitoneal lymph node dissection in patients with non-seminomatous testicular tumor in clinical stage I. Eur Urol 10:17-23, 1984.
5. Fosså SD, Ous S, Lien HH, et al: Post-chemotherapy lymph node histology in radiologically normal patients with metastatic nonseminomatous testicular cancer. J Urol 141:557-559, 1989.
6. Williams SD, Birch R, Einhorn LH, et al: Treatment of disseminated germ-cell tumors with cisplatin, bleomycin, and either vinblastine or etoposide. N Engl J Med 316:1435-1440, 1987.
7. Lewis CR, Fossa SD, Mead G, et al: BOP/VIP--a new platinum-intensive chemotherapy regimen for poor prognosis germ cell tumours. Ann Oncol 2:203-211, 1991.
8. Fossa SD, Droz JP, Stoter G, et al: Cisplatin, vincristine and ifosphamide combination chemotherapy of metastatic seminoma: results of EORTC trial 30874. EORTC GU Group. Br J Cancer 71:619-624, 1995.
9. de Wit R, Roberts JT, Wilkinson PM, et al: Equivalence of three or four cycles of bleomycin, etoposide, and cisplatin chemotherapy and of a 3- or 5-day schedule in good-prognosis germ cell cancer: a randomized study of the European Organization for Research and Treatment of Cancer Genitourinary Tract Cancer Cooperative Group and the Medical Research Council. J Clin Oncol 19:1629-1640, 2001.
10. Kaye SB, Mead GM, Fossa S, et al: Intensive induction-sequential chemotherapy with BOP/VIP-B compared with treatment with BEP/EP for poor-prognosis metastatic nonseminomatous germ cell tumor: a Randomized Medical Research Council/European Organization for Research and Treatment of Cancer study. J Clin Oncol 16:692-701, 1998.
11. Dearnaley DP, Fossa SD, Kaye SB, et al: Adjuvant bleomycin, vincristine and cisplatin (BOP) for high-risk stage I non-seminomatous germ cell tumours: a prospective trial (MRC TE17). Br J Cancer 92:2107-2113, 2005.
12. Cullen MH, Stenning SP, Parkinson MC, et al: Short-course adjuvant chemotherapy in high-risk stage I nonseminomatous germ cell tumors of the testis: a Medical Research Council report. J Clin Oncol 14:1106-1113, 1996.
13. Tandstad T, Dahl O, Cohn-Cedermark G, et al: Risk-adapted treatment in clinical stage I nonseminomatous germ cell testicular cancer: the SWENOTECA management program. J Clin Oncol 27:2122-2128, 2009.

**Supplementary Fig. 1: Flow chart**

Supplementary Fig. 2: Scatterplot showing the association between age and total testosterone and between age and free testosterone
